# Anti‐Inflammatory Peptides as Promising Therapeutics Agent Against Inflammatory Bowel Diseases: A Systematic Review

**DOI:** 10.1002/jgh3.70212

**Published:** 2025-06-29

**Authors:** Kiarash Ghazvini, Zahra Taghiabadi, Mohammad Ali Karimi, Mahdi Hosseini Bafghi, Mahdiesadat Paryan, Razieh Amirfakhrian

**Affiliations:** ^1^ Antimicrobial Resistance Research Center, Department of Microbiology and Virology Faculty of Medicine, Mashhad University of Medical Sciences Mashhad Iran; ^2^ Department of Microbiology and Virology of Medicine Mashhad University of Medical Sciences Mashhad Iran; ^3^ Department of Laboratory Sciences Faculty of Paramedical and Rehabilitation Sciences, Mashhad University of Medical Sciences Mashhad Iran; ^4^ Student Research Committee, Mashhad University of Medical Sciences Mashhad Iran

**Keywords:** anti‐inflammatory peptides, antimicrobial peptide, Crohn's disease, inflammatory bowel diseases, ulcerative colitis

## Abstract

**Background:**

Inflammatory bowel disease (IBD) is linked to dysregulated mucosal immunity, microbiota imbalances, and environmental factors, though its exact cause remains unknown. Current treatments often have limitations, necessitating innovative therapies. This review evaluates anti‐inflammatory peptides (AIPs) as emerging therapeutic agents, focusing on their efficacy in Ulcerative Colitis and Crohn's disease.

**Methodology:**

A systematic review was conducted in February 2023, adhering to PRISMA 2020 guidelines. Studies published from 2010 to 2023 on AIPs for IBD treatment were retrieved from Medline, Web of Science, and Cochrane databases using keywords such as IBDs, AIPs, Crohn's disease, Ulcerative Colitis, and therapy.

**Results:**

Seventeen studies met the inclusion criteria, comprising 12 animal studies, four clinical trials, and one case–control study. H‐SN1 (snake venom peptide) and GLP‐2② (glucagon‐like peptide‐2 dimer) effectively inhibited TNF cytotoxicity. Oral AVX‐470 (bovine‐derived anti‐TNF antibody) reduced enterocyte TNF, MPO, and apoptosis levels. Ac2‐26 (annexin A1 mimic) and αs2‐casein peptide combined with synbiotics were shown to restore gut homeostasis and dysbiosis. AMP‐18 (gastrokine‐1) and MBCP (buffalo milk peptide) stabilized tight junctions, preserving intestinal barrier integrity and potentially preventing IBD progression.

**Conclusion:**

AIPs effectively reduce inflammation, regulate gut microbiota, and stabilize the intestinal barrier, showing promise for managing IBD. However, their therapeutic potential is limited by protease degradation, poor bioavailability, and possible cytotoxicity. Future research should enhance their stability, delivery systems, and pharmacokinetic properties to optimize their clinical applicability and safety.

## 1 Introduction

Inflammatory bowel diseases (IBD) are gastrointestinal chronic inflammation that comprises ulcerative colitis (UC) and Crohn's disease (CD) [1, 2, 3]. IBD is a recurrent inflammatory disorder that, in CD, can cause inflammation of any part of the intestine, particularly the distal ileum, cecum, perianal, as well as colon, whereas UC only affects the rectum and colon [4, 5]. IBD is still an idiopathic disorder; however, some studies revealed that IBD pathogenesis is probably related to the dysregulation of gut mucosal immunity, intestinal microbiota dysbiosis, and genetic and environmental risk factors [5, 6, 7, 8]. Colonic lesions in patients show overexpression of inflammatory mediators that trigger recruitment and chemotaxis of PMNs and lymphocytes [9, 10].

Studies display that Th‐17 cells, innate lymphoid cells (ILCs) [10], as well as TNF‐α, interleukin‐1*β*, interferon‐*γ*, and IL‐23 play a significant role in IBD progression [5, 11, 12]. Targeted conventional therapies involve amino‐salicylates (5‐ASA), azathioprine, corticosteroids, as well as monoclonal antibodies and inhibitors of TNF that could slow down IBD progress, which ranges from mild to severe [13, 14]. Specific side effects accompany the management of IBD with these medicines and are effective only in a fraction of patients [14, 15]. IBD's incidence and prevalence are also rising as the CDC reported that about 3.1 million persons in the USA are affected by these idiopathic colorectal disorders; there needs call for novel therapeutic strategies [16].

Antimicrobial peptides (AMPs) with broad‐spectrum activities act as endogenous antibiotics and provide a new way to fight against infection in serious drug resistance [17, 18, 19]. AMPs comprise approximately 12–50 amino acids [20], exist widely in nature, and are derived from species ranging from bacteria, herbals, and animals to mammals like humans [20]. Their anti‐inflammatory effect complements the antimicrobial properties of AMPs. These cationic polypeptides are involved in the first line of the innate immune system and are commonly known as “host defense peptides” expressed and synthesized by immune cells [21, 22]. Their immunomodulatory activities are varied and specific to AMP types. They include multiple cytokines and growth factor‐like influences to regulate immunity and constitute a linkage between adaptive and innate immune responses [23]. Therefore, the prospect of anti‐inflammatory drugs based on anti‐inflammation peptides has a promising future despite their optimal drug delivery role.

This study systematically reviews the latest evidence on anti‐inflammatory peptides (AIPs) emerging as a novel therapeutic approach in IBDs, particularly in UC and CDs.

## 2 Materials and Methods

### 2.1 Search Strategy

**FIGURE 1:**
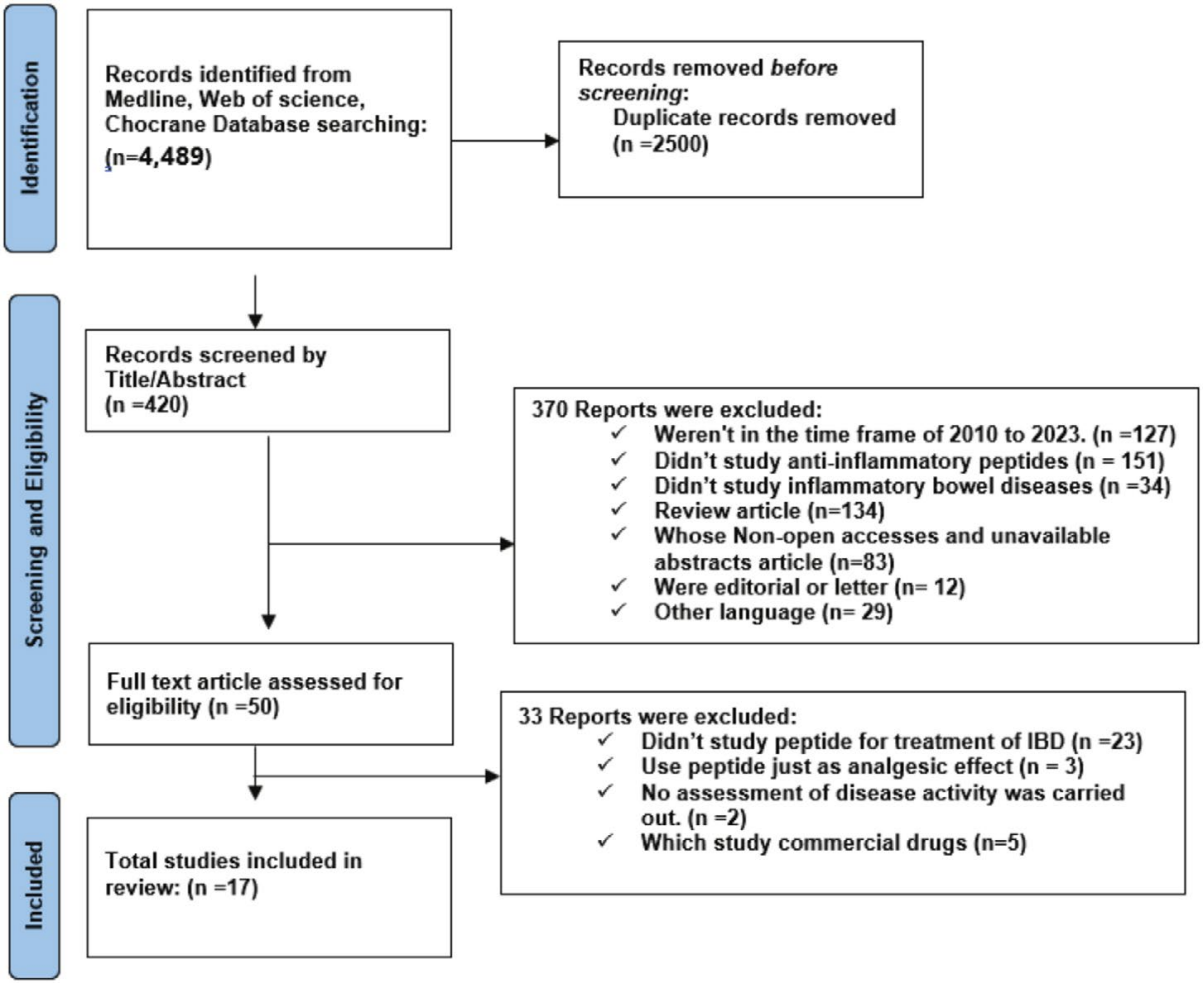
PRISMA 2020 flowchart of study selection.

The present systematic review was carried out in March 2023 following Preferred Reporting Items for Systematic Reviews method recommendations, PRISMA 2020, outlined in Figure 1 [24]. Published studies investigating the use of AIPs for IBD management in patients with colitis and CD were retrieved through the literature in the Medline, Web of Science, and Cochrane databases. Medical Subject heading terms (MeSH) and text words for query were “anti‐inflammatory peptide” AND “inflammatory bowel diseases” OR “IBD” OR “Crohn's disease” OR “Ulcerative colitis” AND “therapy*” AND “treat*.” All duplicate articles were deleted by EndNote (version 20) reference management software. To ensure that all relevant studies were involved, articles selected from the databases were manually and independently reviewed and approved by all authors.

Although the initial search retrieved a large number of studies due to the intentionally broad strategy designed to ensure comprehensive coverage, strict inclusion and exclusion criteria were systematically applied during the screening phases. This approach minimized the risk of missing relevant evidence and ultimately led to a significant reduction in the number of included studies.

### 2.2 Eligibility and Inclusion/Exclusion Criteria

Every study from 2010 to 2023 that used AIPs to eliminate inflammatory bowel progression was considered eligible. Inclusion criteria consist of English and Persian language, original article, mouse and human model, open access article (abstract of nonopen accesses paper were included), the study was published as peer‐reviewed, and those studies which adhere to the research topic. Subsequently, the Exclusion Criteria were as follows: review articles, letters, guidelines, editorials, case reports, and articles unrelated to AIPs.

## 3 Result

### 3.1 Bibliographic and Characteristic of Included Studies

Overall, 17 studies satisfied the predesigned criteria (Figure 1) and were involved in this systematic review, in which 12 reported results used animal models of IBDs, four were clinical trials, and one study was case–control concerning UC and CD patients. Tables 1 and 2 summarize the studies' descriptions, such as types of peptides, their effect on intestinal inflammation, and efficient therapeutic options for moderately to severe IBDs. Four studies included just UC patients, and one investigated patient with IBD. The AIPs used as biologic therapy were peptides B7 and B12 (from *Bifidobacterium longum* and *Bacteroides fragilis*, respectively), Casein glycomacropeptide (CGMP), AVX‐470 (bovine‐derived, antitumor necrosis factor antibody), Anti‐IP‐10 antibody (Interferon‐γ‐inducible protein‐10), Tripeptide K(D)PT.

### 3.2 Anti‐Inflammatory Peptides

#### 3.2.1 In Human Studies

As shown in Table 1, Fernández‐Tomé et al. conducted the first case–control study, which involved 17 active IBD patients (10 CD and seven UC cases) and 20 healthy controls (HCs). Each participant provided two samples: an intestinal biopsy obtained by colonoscopy. At the same time, the other was a blood sample to investigate the presence of cytokines within the mucosal tissues and circulating human antigen‐presenting cells (APCs) phenotype change in the presence or absence of the administered peptides. Peptide B7, derived from probiotic *B. longum* , and peptide B12, derived from *B. fragilis*, were used in this study to determine their effectiveness [25].

The second study by Hvas et al. conducted a randomized pilot study to determine the effectiveness of using CGMP as a nutritional therapy for active UC patients. In this study, 24 active UC patients were chosen and then split into two groups. The first group received the standard treatment alongside consuming 30 g of CGMP/day, while the control group only received the standard treatment (4800 mg oral mesalamine daily) over 1 month. For analysis purposes, biopsy samples, blood samples (to measure circulating cytokines and detect the level of Hb, CRP, WBC count, and albumin), and stool samples (for fecal calprotectin assessment) were collected from both groups [26].

Hartman et al. conducted a clinical trial (NCT01759056) to evaluate the efficacy of AVX‐470, a bovine‐derived anti‐TNF antibody, in treating active UC. The study involved 36 patients randomly assigned to receive AVX‐470 at three doses (0.2, 1.6, or 3.5 g/day) or a placebo for 1 month. The study collected biopsy samples from five bowel segments to assess tissue inflammatory biomarkers using immunohistochemical staining and qRT‐PCR. These biomarkers included anti‐TNF, antibovine Ig, anti‐IL‐1β, anti‐CD3, anti‐CD68, and antimyeloperoxidase (MPO). Additionally, epithelial cell apoptosis was evaluated using TUNEL staining. The researchers aimed to determine whether AVX‐470 could reduce inflammation in the gut tissue of UC patients by targeting TNF. This proinflammatory cytokine plays a critical role in the development and progression of UC. They measured tissue biomarkers to assess the drug's effectiveness in reducing inflammation and TUNEL staining to evaluate its effect on cell death in the gut lining. However, it was observed that drug selectivity and stability were one of the major problems of this study [27].

In a double‐blind phase II randomized study, Mayer and colleagues (trial identifier: NCT00656890) evaluated the potential effectiveness of an anti‐IP‐10 antibody as a therapy for UC. The antibody targets Interferon‐γ‐inducible protein‐10. 109 patients with UC were enrolled, with 55 patients receiving the peptide and 54 receiving a placebo. Patients received 10 mg/kg of the anti‐IP‐10 antibody biweekly for 8 weeks. The researchers assessed the clinical response rate at day 57 by measuring reductions in the Mayo total score by three or more points and a decrease in the rectal bleeding score by one or more points. They also evaluated the mucosal healing rates, which required a Mayo total score of two or less with no individual score more significant than one, and total clinical remission was described as a Mayo total endoscopic score of one or less. In addition, the researchers conducted post hoc analyses to evaluate drug exposure‐response association and histological amelioration. Overall, the study aimed to determine whether the anti‐IP‐10 antibody could be an effective treatment for improving clinical symptoms and mucosal healing in UC patients [28].

The study conducted by Kucharzik and colleagues was a phase 2 clinical trial designed to investigate the efficacy of K(D)PT medication in UC cases. The trial was double‐blind, randomized, and lasted for 8 weeks. Patients were given oral K(D)PT twice daily at three doses (20, 50, or 100 mg). The researchers assessed the drug's effectiveness by measuring the colitis activity index (CAI) after treatment. They also evaluated the rate of improvement at different time points during the study. The CAI score indicates the severity of inflammation in the colon, with higher scores indicating more severe symptoms. Therefore, the researchers were interested in whether K(D)PT could reduce CAI scores and improve the rate of improvement in UC patients. In this study, the researchers also aimed to increase the drug's tolerability and find ways for the drug to overcome existing problems in this area [29].

**TABLE 1:** Study characteristics of AIPs as therapeutic agents in IBD patients.

No.	Author	Study design	Patient	IBD type	Peptide	Assess inflammatory markers	Outcome
1	Fernández‐Tomé et al. [25]	Case–control	Eight IBD patients/10 healthy controls	IBD	*Bifidobacterium longum* (peptide B7) *Bacteroides fragilis* (peptide B12)	The mucosal tissue cytokine of IBD patients didn't change by these peptides. The peptide B7 decreased the expression of CCR2 on APC in healthy controls but not in IBD patients. However, peptide B12 didn't have the same effect.	These peptides were not reversing the altered mucosal cytokine seen in IBD patients and may not be effective treatments for IBD.
2	Hvas et al. [26]	Randomized pilot study	24 patients	UC	Casein glycomacropeptide (CGMP)	NA^a^	Have good safety. The impact of CGMP on IBD modification was comparable to increasing the dose of mesalamine.
3	Hartman et al. [27]	Randomized controlled trial	36 patients	UC	AVX‐470 (bovine‐derive, anti‐TNF antibody)	Anti‐TNF activity by more than 10‐fold TNF decreases and reduces MPO and apoptosis levels in enterocytes.	Oral administration of 0.2, 1.6, or 3.5 g/day was effective in UC cases.
4	Mayer et al. [28]	Phase II randomized clinical trial study	109 patients (*n* = 55; placebo: *n* = 54)	UC	Anti‐IP‐10 antibody (BMS‐936557)	NA	Anti‐IP‐10 (10 mg/kg) was a beneficial option for colitis treatment.
5	Kucharzik et al. [29]	Randomized multicenter study	NA	UC	Tripeptide K(D)PT	NA	K(D)PT was a practical option for colitis treatment and showed an excellent safety profile.

^a^ Not assessed.

Due to the high costs and significant risks associated with human trials, animal studies have garnered considerable attention from scientists [42]. Many studies highlight the remarkable functional similarities between humanized mice and humans, making these mice a common choice for in vivo research [43]. We have reviewed several studies on AIPs using humanized mice models in this context.

#### 3.2.2 In Animal Model Studies

A wide range of AIPs in IBD's animal models was analyzed in this systematic review, such as Vasoactive Intestinal Peptide, Thrombospondin peptide ABT‐898, Self‐assembling Peptide Hydrogel (SAPH, PuraMatrix), glucagon‐like peptide‐2 dimer (GLP‐2②), Hydrostatin‐SN1 (H‐SN1), Cortistatin (A5), *α*s2‐casein, peptide 317, Peptide drove from annexin A1, annexin A1‐mimetic peptide Ac2‐26, *Bubalus bubalis* milk‐derived products (MBCP), AMP‐18 (Antrum Mucosal Protein).

**TABLE 2:** Study characteristics of AIPs as therapeutic agents in IBD's animal models.

No.	Author	Study design	Animal model	Peptide	IBD type	Assess inflammation marker	Outcome
1	Jayawardena et al. [30]	In vivo	Mice	Vasoactive intestinal peptide	DSS‐induced colitis^a^	The expression of mRNA in proinflammatory cytokines was decreased.	They were influential in colitis treatment in mice and displayed high histological improvement.
2	Gutierrez et al. [31]	In vivo	Mice	Thrombospondin peptide ABT‐898	DSS_induced colitis	IL‐6 and positive cells for signal transducer and activator of transcription 3 (STAT3) were decreased.	Peptide ABT‐898 reduced inflammatory response and angiogenesis and may be effective in IBD.
3	Araki et al. [32]	In vivo	Rat	Self‐assembling Peptide Hydrogel (SAPH, PuraMatrix)	TNBS‐induced colitis	Reduced colonic overexpression of *IL‐1*α and *IL‐6*	Successfully reduced colonic damage, inflammatory cytokine overexpression, and enhanced wound healing
3	J Gu et al. [33]	In vitro/In vivo	BALB/c mice	Glucagon‐like peptide‐2 dimer (GLP‐2②)	DSS‐induced colitis	Decreased the MPO and protein expression of NLRP3 and COX2 in the colonocyte.	Have antiapoptosis activity in the colon and is an effective option for IBD management.
4	Zheng et al. [34]	In vitro/in vivo	Mice	Hydrostatin‐SN1 (H‐SN1)	DSS‐induced acute colitis	Suppressed the expression of mRNA of TNF/TNFR1	Alleviate the colitis clinical indicators such as disease activity index and histologic scores
5	Rol et al. [35]	In vitro/in vivo	Mice	Cortistatin (A5)	IBD	NA^b^	demonstrated an extended half‐life in the bloodstream and exhibited a distinct receptor binding profile.
6	J Ha et al. [36]	in vivo	Mice	*α*s2‐casein (a peptide derived from the Synbiotics, Fermented *Cudrania tricuspidata* with *Lactobacillus gasseri* )	DSS‐induced IBD	Decreased the overexpression of IL‐1*β*, IL‐6, TNF‐*α*, and COX‐2 in the IBD patients.	Pep 2 extracted from αs2‐casein was beneficial in suppressing IBD's inflammation.
7	Sobczak et al. [37]	In vivo	Mice	Peptide 317 (analog of opioid peptide morphiceptin)	UC/CD	Reduced in the expression of proinflammatory cytokines mRNA	Administration P‐317 in 0.1 mg/kg showed anti‐inflammatory and antinociceptive influence.
8	Caceres et al. [38]		Mice	MC‐12 (Peptide drived from annexin A1)	TNBS‐induced colitis^c^	NA	Cyclic peptides could be effective in IBD therapy.
9	Li et al. [39]	In vitro/in vivo	Mice	AON (annexin A1‐mimetic peptide Ac2‐26)	DSS‐induced colitis	Decreased inflammation signs	Have good safety in oral administration and could be effective in IBD therapy.
10	Tenore et al. [40]	In Caco2 cells culture (in vitro)/in vivo	Mice	MBCP ( *Bubalus bubalis* milk‐derived products)	DNBS‐induced colitis^d^	Stabilized tight junctions regulate the nuclear factor (NF)‐κB pathway	Aid to repair the intestinal barrier, which is disturbed via inflammation
11	Chen et al. [41]	In vitro/ex vivo	Mice	AMP‐18 (Antrum Mucosal Protein)	NSAID^e^‐induced colitis	NA	Plays a defensive role in preventing injury along the GI mucosal barrier.

^a^ Dextran sulfate sodium (DSS)‐induced colitis.

^b^ Not assessed.

^c^ Trinitrobenzenesulfonic acid (TNBS).

^d^ Dinitrobenzene sulfonic acid (DNBS).

^e^ Nonsteroidal anti‐inflammatory drug (NSAID).

Two recent studies have highlighted the anti‐inflammatory potential of peptides derived from *Hericium erinaceus*, a medicinal mushroom. An ex vivo study demonstrated that these peptides can significantly attenuate inflammation in human IBD models [44]. Furthermore, a clinical study published in 2024 confirmed their beneficial effects in patients with UC [45]. These findings suggest that *H. erinaceus*‐derived peptides could be promising therapeutic candidates for IBD management.

## 4 Discussion

The pathogenesis of IBD is closely linked to the presence of inflammatory cytokines, including IL‐1β, IFN‐γ, TNF‐α, and Interleukin‐6/10/18/33. These cytokines play a critical role in initiating, progressing, and resolving inflammation in the gut. Still, they can also cause tissue damage and sometimes perpetuate the disease. Their proinflammatory effects can lead to the destruction of intestinal epithelial cells, which further exacerbates inflammation in the affected area. It is essential to monitor the levels of these cytokines in patients with IBD to manage their symptoms effectively. Furthermore, developing therapies targeting these cytokines may hold promise for more effective treatment options in the future [25, 46, 47]. Therefore, the current treatment methods for IBD involve immunomodulators and immunosuppressives. Examples of these treatments include Azathioprine, Ciclosporin, anti‐integrin agents such as Vedolizumab, anti‐interleukin agents like Ustekinumab, and anti‐TNFα antibodies, which encompass Infliximab, Adalimumab, and Golimumab. These therapies work by modulating or suppressing the immune system to reduce inflammation in the gut and alleviate symptoms associated with IBD. However, it is essential to note that these treatments may have side effects and may not work for everyone, highlighting the need for continued research into alternative therapeutic options [48, 49].

**FIGURE 2:**
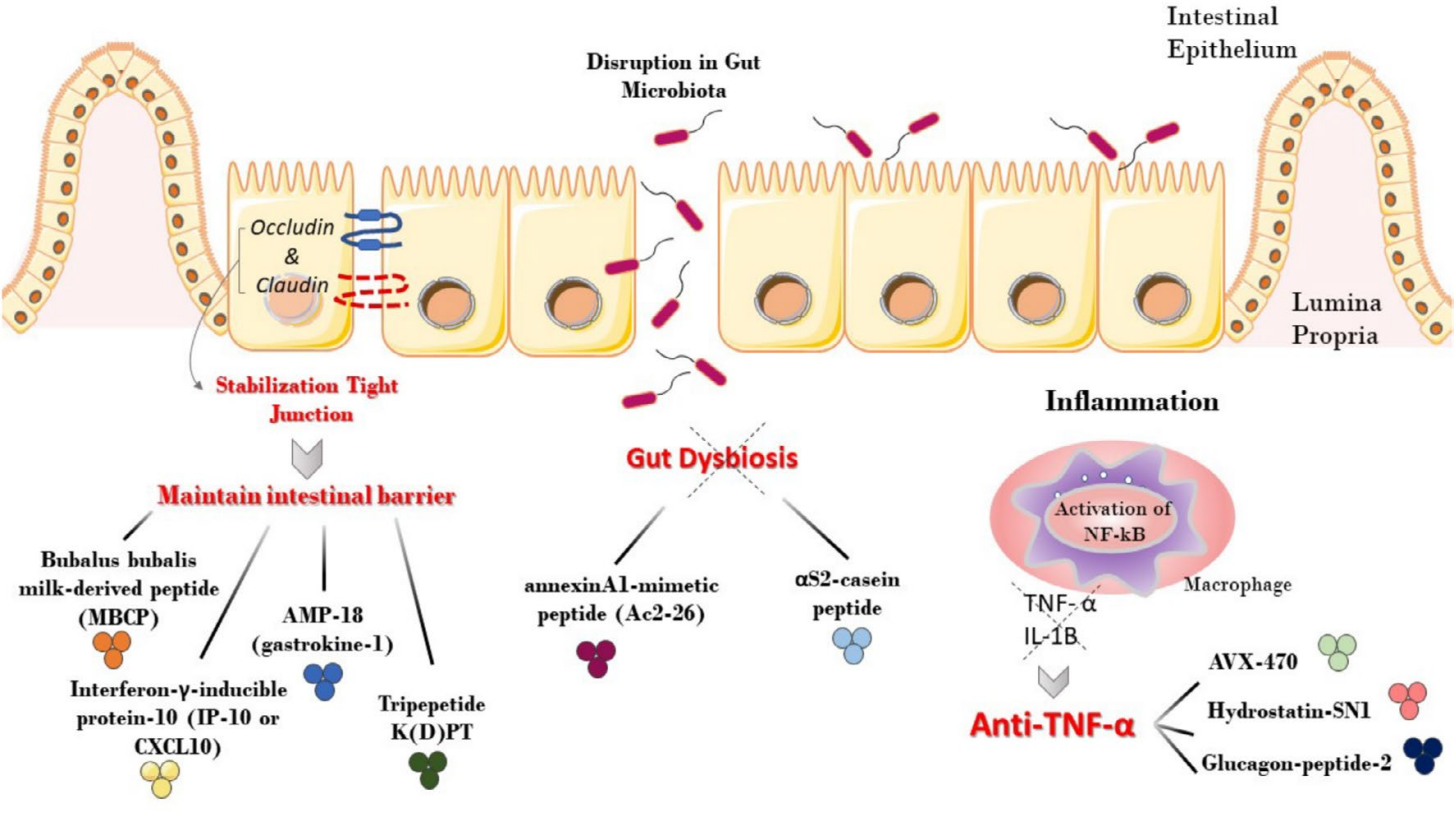
AIPs are effective in IBD treatment through three mechanisms of action. MBCP ( *Bubalus bubalis* milk‐derived products), Anti‐IP‐10 antibody (Interferon‐γ‐inducible protein‐10), AMP‐18 (Gastrokine‐1), and Tripeptide K(D)PT play a role in intestinal barrier maintenance through stabilization of Tight Junction. Another two peptides, annexinA1‐mimetic peptide (Ac2‐26) and αS2‐casein peptide, help the maintenance of Gut Microbiota. AVX‐470, Hydrostatin‐SN1 and glucagon‐like peptide‐2 dimer (GLP‐2②) has anti‐TNF activity.

In UC and CD cases, anti‐TNF agents have proven highly efficient and had significant breakthroughs in IBD treatment [50, 51]. Studies have shown that H‐SN1, a natural peptide derived from the venom of *Hydrophis cyanocinctus* snakes, significantly inhibits tumor necrosis factor cytotoxicity in L929 fibroblast cells. According to Zheng et al., this peptide is capable of binding to TNF‐α receptor 1 (TNFR1), which leads to the suppression of TNF/TNFR1 axis signaling and inhibition of NF‐кB pathways and MAPK activation in HEK293 embryonic kidney and HT29 adenocarcinoma cell lines. Moreover, experiments conducted on mice using the DSS‐induced colitis model indicated that the anti‐inflammatory properties of H‐SN1 effectively reduced the clinical symptoms of colitis, including decreased body weight loss, disease severity, bloody diarrhea, and colon inflammation. These findings suggest that H‐ SN1 could be the prospective therapy for IBD [34]. H‐SN1 as a treatment method resulted in a notable decrease in proinflammatory cytokines, such as IL‐1β, IFN‐γ, and IL‐6 transcripts. These results were similar to those achieved using GLP‐2②, a potent candidate for IBD therapy. Both H‐SN1 and GLP‐2② have been found to effectively reduce these proinflammatory cytokine levels, which are commonly involved in the onset and progression of IBD [33, 34]. The gut hormone GLP‐2, known for promoting intestinal growth, demonstrated promising results in a murine model of colitis treatment. The treated mice showed increased body weight and reduced colitis scores comparable to those of the other peptide. Additionally, GLP‐2 was found to alleviate MPO activities and decrease protein expression of NLR Family Pyrin Domain Containing 3 (NLRP3) and Cyclooxygenase 2 (COX2), which are both associated with inflammation [33]. Moreover, when AVX‐470 (bovine‐derived, anti‐TNF antibody) was administered orally at a dosage of 3.5 g/day for 4 weeks, it reduced TNF, MPO, and apoptosis levels in enterocytes observed in biopsy specimens. This treatment was found to be effective in patients who were suffering from active UC [27] (Figure 2).

Disruptions in the gut microbiota might be associated with IBD progression. Specifically, dysbiosis can negatively impact the function of the intestinal barrier. This can lead to changes in the secretion of AMPs and mucus glycosylation, which are essential for supporting healthy gut bacteria growth. Additionally, these changes can create an environment where commensal bacteria living in the gut cannot properly adhere to and function as they should [52, 53]. In addition to the current immune‐suppressing treatment for patients with IBD, novel biologic therapies focused on maintaining gut hemostasis and reversing gut dysbiosis could be effective [54]. The use of Ac2‐26, a peptide that mimics annexin A1, in experimental colitis models has been found to cause changes in the makeup of the intestinal microbiota. Additionally, this peptide has been observed to boost the production of short‐chain fatty acids (SCFAs), which have essential roles in maintaining gut health and reducing inflammation. Therefore, Ac2‐26 shows promise as a potential therapeutic agent for treating colitis and related conditions [39]. A study conducted by Li and colleagues found that mice with colitis induced by dextran sulfate sodium (DSS) experienced a reduction in the abundance of Escherichia‐Shigella bacteria when treated with oral administration of AON (Ac2‐26, which is a type of oxidation‐labile Nanotherapy). These particular bacterial species have been associated with the development of colitis and other IBDs. Therefore, reducing their abundance may be beneficial in managing colitis symptoms and improving overall gut health. The findings suggest that treatment with AON can manipulate the gut microbiome in a way that promotes a more balanced and diverse gut ecosystem, which can, in turn, help prevent the development of inflammatory diseases like colitis [55]. Furthermore, there was an increase in the prevalence of certain bacterial species belonging to the Prevotellaceae family in the colitis group. These particular bacteria are known to produce SCFAs, which are the preferred source of energy for cells in the colon. SCFAs have anti‐inflammatory properties and help to maintain a healthy balance of gut bacteria. This suggests that even though colitis is characterized by inflammation, the body may try to counteract this by increasing the abundance of bacteria that produce anti‐inflammatory compounds like SCFAs. Overall, this finding highlights the critical role that gut bacteria play in maintaining gut health and preventing inflammatory diseases [39]. SCFA compounds have several vital functions within the body, including immune modulation. Specifically, SCFAs can decrease the release of proinflammatory cytokines via macrophages. This is achieved by activating G protein receptors known as GPR43 and inhibiting an enzyme called histone deacetylase. As a result, they may be beneficial in treating inflammatory conditions like IBD [56]. Similarly, taking synbiotics, a combination of probiotics and prebiotics, can be advantageous in treating IBD because they can help decrease the expression of cytokines that promote inflammation. Essentially, synbiotics work by introducing beneficial bacteria into the gut, along with the fibers that feed these bacteria. This helps to balance the microbiome in the gut, decreasing the abundance of opportunistic bacteria that can trigger inflammation and increasing the presence of beneficial bacteria that can help to reduce inflammation. As a result, synbiotic supplements may help manage symptoms of IBD and improve overall gut health [57]. A study by Ha et al. investigated the use of a peptide called αs2‐casein to treat mice with IBD. This study found that using this peptide decreased inflammation associated with IBD. It is worth noting that this peptide was isolated from synbiotics, a combination of fermented *Cudrania tricuspidata* extract and *Lactobacillus gasseri* . This suggests combining the αs2‐casein peptide with synbiotics may effectively treat IBD‐related inflammation [36]. However, two types of peptides, specifically those derived from gut commensals *B. longum* (known as peptide B7) and opportunistic bacteria *B. fragilis* (known as peptide B12), were not successful in reversing the altered mucosal cytokine profile seen in individuals with IBD. This suggests that these peptides may not be effective treatments for IBD [25].

Melanocortin‐derived peptides, including α‐MSH and its synthetic analogs, have shown substantial anti‐inflammatory and immunomodulatory potential. These peptides modulate cytokine profiles, preserve epithelial integrity, and regulate innate immune responses in experimental models of IBD [58]. Their promising biological activities highlight their value as emerging candidates in peptide‐based IBD therapies.

Some recent studies have used animal models to investigate the connection between an impaired intestinal barrier and the progression of IBD. The research suggests that stabilizing tight junctions (TJs) in the intestine may effectively prevent IBD. This indicates that there may be a link between the breakdown of the gastrointestinal barrier and the pathogenesis or development of IBD [59, 60]. When mice with colitis were given a specific type of AMP called AMP‐18 (also known as gastrokine‐1), it positively affected their intestinal health. Specifically, it helped to reverse the hyper‐permeability (or increased permeability) of the gastrointestinal mucosal barrier that is often seen in cases of colitis. This was achieved by stabilizing the TJs in the colon's epithelial tissue. By doing so, the wound‐healing process in the affected area was accelerated. Overall, this suggests that AMP‐18 may be a promising treatment option for individuals suffering from colitis [41]. Research has shown that a peptide derived from buffalo milk, called MBCP, may have the ability to repair damage to the intestinal epithelium caused by inflammation related to IBD. Doing so helps maintain the integrity of the intestinal barrier. In laboratory experiments using Caco‐2 cells induced with TNF‐α—a substance known to cause inflammation—MBCP was found to be effective in regulating the activity of the NF‐κB pathway, which is involved in immune responses and inflammation. Additionally, it was observed to reduce intestinal permeability in mice models. Consequently, MBCP could be a prospective therapeutic agent for individuals with IBD who experience damage to the intestinal barrier [40]. Furthermore, a clinical trial study has also explored the use of AIPs. One such peptide is Interferon‐γ‐inducible protein‐10 (IP‐10 or CXCL10), used in a phase II randomized clinical trial for patients with moderate‐to‐severe UC. The trial demonstrated that IP‐10 increased these patients' mucosal healing rates and histological improvement. This suggests that IP‐10 has the potential to modulate epithelial homeostasis (balance) and could, therefore, be a promising treatment option for individuals with IBD [28]. Meanwhile, anti‐inflammatory tripeptide K(D)PT was efficient in UC patients, probably due to the fixation of TJ protein [29].

Despite the promising findings, several research gaps remain for many of the peptides discussed in this review. Key limitations include a lack of pharmacokinetic data, insufficient large‐scale human trials, and unclear long‐term safety profiles. A summary of each peptide's observed effects and the corresponding research gaps is presented in (Table S1).

## 5 Conclusion

The present study explored the potential of AIPs in treating IBD and their clinical benefits. The findings indicate that AIPs could serve as a viable alternative or complement to current therapeutic strategies. These peptides modulate the immune response, reduce inflammation, maintain mucosal homeostasis, regulate gut microbiota, and reinforce the intestinal barrier. However, their clinical application remains limited due to challenges such as susceptibility to proteolytic degradation and potential cytotoxicity. Therefore, future research should aim to improve the pharmacokinetic properties of these peptides, focusing on enhancing their stability, specificity, and targeted drug delivery mechanisms to optimize their therapeutic efficacy in IBD treatment.

## Ethics Statement

The authors have nothing to report.

## Consent

The authors have nothing to report.

## Conflicts of Interest

The authors declare no conflicts of interest.

## Supporting information


**Data S1.** Supporting Information.

## Data Availability

All data generated or analyzed in this study are included in this article.

## References

[1] M. E. Kuenzig , S. G. Fung , L. Marderfeld , et al., “Twenty‐First Century Trends in the Global Epidemiology of Pediatric‐Onset Inflammatory Bowel Disease: Systematic Review,” Gastroenterology 162 (2022): 1147–1159. 34995526 10.1053/j.gastro.2021.12.282

[2] R. Sigala‐Robles , L. Santiago‐López , A. Hernández‐Mendoza , et al., “Peptides, Exopolysaccharides, and Short‐Chain Fatty Acids From Fermented Milk and Perspectives on Inflammatory Bowel Diseases,” Digestive Diseases and Sciences 67, no. 10 (2022): 4654–4665. 35133532 10.1007/s10620-022-07382-2

[3] C. Dai , Y. H. Huang , and M. Jiang , “Combination Therapy in Inflammatory Bowel Disease: Current Evidence and Perspectives,” International Immunopharmacology 114 (2023): 109545. 36508920 10.1016/j.intimp.2022.109545

[4] W.‐s. Yang , J.‐l. Wang , W. Wu , et al., “Formyl Peptide Receptor 2 as a Potential Therapeutic Target for Inflammatory Bowel Disease,” Acta Pharmacologica Sinica 44, no. 1 (2022): 19–31. 35840658 10.1038/s41401-022-00944-0 PMC9812994

[5] Q. Guan , “A Comprehensive Review and Update on the Pathogenesis of Inflammatory Bowel Disease,” Journal of Immunology Research (2019): 7247238. 31886308 10.1155/2019/7247238 PMC6914932

[6] P. T. Santana , S. L. B. Rosas , B. E. Ribeiro , Y. Marinho , and H. S. P. de Souza , “Dysbiosis in Inflammatory Bowel Disease: Pathogenic Role and Potential Therapeutic Targets,” International Journal of Molecular Sciences 23, no. 7 (2022): 3464. 35408838 10.3390/ijms23073464 PMC8998182

[7] K. G. Upadhyay , D. C. Desai , T. F. Ashavaid , and A. J. Dherai , “Microbiome and Metabolome in Inflammatory Bowel Disease,” Journal of Gastroenterology and Hepatology 38, no. 1 (2023): 34–43. 36287112 10.1111/jgh.16043

[8] M. H. Bafghi , F. Ghanipour , R. Nazari , S. S. Aghaei , and P. Jafari , “Enhancing the Antibacterial Impact of Lipopeptide Extracted From *Bacillus Licheniformis* as a Probiotic Against MDR *Acinetobacter Baumannii* ,” Frontiers in Bioscience‐Landmark 29, no. 5 (2024): 171. 10.31083/j.fbl2905171 38812307

[9] N. A. Nasef and S. Mehta , “Role of Inflammation in Pathophysiology of Colonic Disease: An Update,” International Journal of Molecular Sciences 21, no. 13 (2020): 4748. 32635383 10.3390/ijms21134748 PMC7370289

[10] S. H. Lee , J. E. Kwon , and M. L. Cho , “Immunological Pathogenesis of Inflammatory Bowel Disease,” Intestinal Research 16, no. 1 (2018): 26–42. 29422795 10.5217/ir.2018.16.1.26 PMC5797268

[11] M. F. Neurath , “IL‐23 in Inflammatory Bowel Diseases and Colon Cancer,” Cytokine & Growth Factor Reviews 45 (2019): 1–8. 30563755 10.1016/j.cytogfr.2018.12.002

[12] B. Verstockt , A. Salas , B. E. Sands , et al., “IL‐12 and IL‐23 Pathway Inhibition in Inflammatory Bowel Disease,” Nature Reviews. Gastroenterology & Hepatology 20, no. 7 (2023): 433–446. 37069321 10.1038/s41575-023-00768-1 PMC10958371

[13] G. Privitera , D. Pugliese , S. Onali , et al., “Combination Therapy in Inflammatory Bowel Disease – From Traditional Immunosuppressors Towards the New Paradigm of Dual Targeted Therapy,” Autoimmunity Reviews 20, no. 6 (2021): 102832. 33866066 10.1016/j.autrev.2021.102832

[14] T. Larussa , M. Imeneo , and F. Luzza , “Potential Role of Nutraceutical Compounds in Inflammatory Bowel Disease,” World Journal of Gastroenterology 23, no. 14 (2017): 2483–2492. 28465632 10.3748/wjg.v23.i14.2483 PMC5394511

[15] Z. Cai , S. Wang , and J. Li , “Treatment of Inflammatory Bowel Disease: A Comprehensive Review,” Frontiers in Medicine 8 (2021): 765474. 34988090 10.3389/fmed.2021.765474 PMC8720971

[16] F. Xu , J. M. Dahlhamer , E. P. Zammitti , A. G. Wheaton , and J. B. Croft , “Health‐Risk Behaviors and Chronic Conditions Among Adults With Inflammatory Bowel Disease—United States, 2015 and 2016,” MMWR. Morbidity and Mortality Weekly Report 67, no. 6 (2018): 190–195. 29447146 10.15585/mmwr.mm6706a4 PMC5815485

[17] J. Sun , P. Kong , J. Shi , and Y. Liu , “Evaluation of the Antibacterial Potential of Two Short Linear Peptides YI12 and FK13 Against Multidrug‐Resistant Bacteria,” Pathogens 13, no. 9 (2024): 797. 39338988 10.3390/pathogens13090797 PMC11435022

[18] Y. Zhu , W. Hao , X. Wang , et al., “Antimicrobial Peptides, Conventional Antibiotics, and Their Synergistic Utility for the Treatment of Drug‐Resistant Infections,” Medicinal Research Reviews 42, no. 4 (2022): 1377–1422. 34984699 10.1002/med.21879

[19] Y. Haitao , C. Yifan , S. Mingchao , and H. Shuaijuan , “A Novel Polymeric Nanohybrid Antimicrobial Engineered by Antimicrobial Peptide MccJ25 and Chitosan Nanoparticles Exerts Strong Antibacterial and Anti‐Inflammatory Activities,” Frontiers in Immunology 12 (2021): 811381. 35126369 10.3389/fimmu.2021.811381 PMC8807516

[20] G. Satchanska , S. Davidova , and A. Gergova , “Diversity and Mechanisms of Action of Plant, Animal, and Human Antimicrobial Peptides,” Antibiotics 13, no. 3 (2024): 202. 38534637 10.3390/antibiotics13030202 PMC10967526

[21] H. Li , J. Niu , X. Wang , M. Niu , and C. Liao , “The Contribution of Antimicrobial Peptides to Immune Cell Function: A Review of Recent Advances,” Pharmaceutics 15, no. 9 (2023): 2278. 37765247 10.3390/pharmaceutics15092278 PMC10535326

[22] M. Hemshekhar , V. Anaparti , and N. Mookherjee , “Functions of Cationic Host Defense Peptides in Immunity,” Pharmaceuticals 9, no. 3 (2016): 40. 27384571 10.3390/ph9030040 PMC5039493

[23] P. Chieosilapatham , F. Niyonsaba , C. Kiatsurayanon , K. Okumura , S. Ikeda , and H. Ogawa , “The Antimicrobial Peptide Derived From Insulin‐Like Growth Factor‐Binding Protein 5, AMP‐IBP5, Regulates Keratinocyte Functions Through Mas‐Related Gene X Receptors,” Journal of Dermatological Science 88, no. 1 (2017): 117–125. 28554590 10.1016/j.jdermsci.2017.05.008

[24] M. Page , J. McKenzie , P. Bossuyt , et al., “The PRISMA 2020 Statement: An Updated Guideline for Reporting Systematic Reviews,” Systematic Reviews 10, no. 1 (2021): 1–11. 33781348 10.1186/s13643-021-01626-4 PMC8008539

[25] S. Fernández‐Tomé , A. C. Marin , L. Ortega Moreno , et al., “Immunomodulatory Effect of Gut Microbiota‐Derived Bioactive Peptides on Human Immune System From Healthy Controls and Patients With Inflammatory Bowel Disease,” Nutrients 11, no. 11 (2019): 2605. 31683517 10.3390/nu11112605 PMC6893616

[26] C. L. Hvas , A. Dige , M. Bendix , et al., “Casein Glycomacropeptide for Active Distal Ulcerative Colitis: A Randomized Pilot Study,” European Journal of Clinical Investigation 46, no. 6 (2016): 555–563. 27090817 10.1111/eci.12634

[27] D. S. Hartman , D. E. Tracey , B. R. Lemos , et al., “Effects of AVX‐470, an Oral, Locally Acting Anti‐Tumour Necrosis Factor Antibody, on Tissue Biomarkers in Patients With Active Ulcerative Colitis,” Journal of Crohn's & Colitis 10, no. 6 (2016): 641–649. 10.1093/ecco-jcc/jjw026 26802087

[28] L. Mayer , W. J. Sandborn , Y. Stepanov , et al., “Anti‐IP‐10 Antibody (BMS‐936557) for Ulcerative Colitis: A Phase II Randomised Study,” Gut 63, no. 3 (2014): 442–450. 23461895 10.1136/gutjnl-2012-303424 PMC3933070

[29] T. Kucharzik , G. Lemmnitz , C. Abels , and C. Maaser , “Tripeptide K(D)PT Is Well Tolerated in Mild‐To‐Moderate Ulcerative Colitis: Results From a Randomized Multicenter Study,” Inflammatory Bowel Diseases 23, no. 2 (2017): 261–271. 28092306 10.1097/MIB.0000000000001000

[30] D. Jayawardena , A. N. Anbazhagan , G. Guzman , P. K. Dudeja , and H. Onyuksel , “Vasoactive Intestinal Peptide Nanomedicine for the Management of Inflammatory Bowel Disease,” Molecular Pharmaceutics 14, no. 11 (2017): 3698–3708. 28991483 10.1021/acs.molpharmaceut.7b00452 PMC6053281

[31] L. S. Gutierrez , J. Ling , D. Nye , K. Papathomas , and C. Dickinson , “Thrombospondin Peptide ABT‐898 Inhibits Inflammation and Angiogenesis in a Colitis Model,” World Journal of Gastroenterology 21, no. 20 (2015): 6157. 26034351 10.3748/wjg.v21.i20.6157 PMC4445093

[32] T. Araki , K. Mitsuyama , H. Yamasaki , et al., “Therapeutic Potential of a Self‐Assembling Peptide Hydrogel to Treat Colonic Injuries Associated With Inflammatory Bowel Disease,” Journal of Crohn's & Colitis 15, no. 9 (2021): 1517–1527. 10.1093/ecco-jcc/jjab033 PMC8464220 33596312

[33] J. Gu , J. Liu , T. Huang , et al., “The Protective and Anti‐Inflammatory Effects of a Modified Glucagon‐Like Peptide‐2 Dimer in Inflammatory Bowel Disease,” Biochemical Pharmacology 155 (2018): 425–433. 30040929 10.1016/j.bcp.2018.07.027

[34] Z. Zheng , H. Jiang , Y. Huang , et al., “Screening of an Anti‐Inflammatory Peptide From *Hydrophis cyanocinctus* and Analysis of Its Activities and Mechanism in DSS‐Induced Acute Colitis,” Scientific Reports 6, no. 1 (2016): 1–13. 27158082 10.1038/srep25672 PMC4860709

[35] Á. Rol , T. Todorovski , P. Martin‐Malpartida , et al., “Structure‐Based Design of a Cortistatin Analogue With Immunomodulatory Activity in Models of Inflammatory Bowel Disease,” Nature Communications 12, no. 1 (2021): 1869. 10.1038/s41467-021-22076-5 PMC7994712 33767180

[36] J. Ha , H. Oh , N. S. Oh , et al., “Anti‐Inflammatory Effect of a Peptide Derived From the Synbiotics, Fermented *Cudrania tricuspidata* With *Lactobacillus gasseri* , on Inflammatory Bowel Disease,” Mediators of Inflammation 2020 (2020): 1–8. 10.1155/2020/3572809 PMC7355370 32714090

[37] M. Sobczak , P. K. Zakrzewski , A. I. Cygankiewicz , et al., “Anti‐Inflammatory Action of a Novel Orally Available Peptide 317 in Mouse Models of Inflammatory Bowel Diseases,” Pharmacological Reports 66, no. 5 (2014): 741–750. 25149976 10.1016/j.pharep.2014.03.007

[38] C. C. Caceres , P. S. Bansal , S. Navarro , et al., “An Engineered Cyclic Peptide Alleviates Symptoms of Inflammation in a Murine Model of Inflammatory Bowel Disease,” Journal of Biological Chemistry 292, no. 24 (2017): 10288–10294. 28473469 10.1074/jbc.M117.779215 PMC5473231

[39] C. Li , Y. Zhao , J. Cheng , et al., “A Proresolving Peptide Nanotherapy for Site‐Specific Treatment of Inflammatory Bowel Disease by Regulating Proinflammatory Microenvironment and Gut Microbiota,” Advanced Science 6, no. 18 (2019): 1900610. 31559126 10.1002/advs.201900610 PMC6755521

[40] G. C. Tenore , E. Pagano , S. Lama , et al., “Intestinal Anti‐Inflammatory Effect of a Peptide Derived From Gastrointestinal Digestion of Buffalo ( *Bubalus bubalis* ) Mozzarella Cheese,” Nutrients 11, no. 3 (2019): 610. 30871183 10.3390/nu11030610 PMC6471453

[41] P. Chen , D. Bakke , L. Kolodziej , et al., “Antrum Mucosal Protein‐18 Peptide Targets Tight Junctions to Protect and Heal Barrier Structure and Function in Models of Inflammatory Bowel Disease,” Inflammatory Bowel Diseases 21, no. 10 (2015): 2393–2402. 26197453 10.1097/MIB.0000000000000499 PMC4567513

[42] A. Kapała , M. Szlendak , and E. Motacka , “The Anti‐Cancer Activity of Lycopene: A Systematic Review of Human and Animal Studies,” Nutrients 14, no. 23 (2022): 5152. 36501182 10.3390/nu14235152 PMC9741066

[43] J. Chuprin , H. Buettner , M. O. Seedhom , et al., “Humanized Mouse Models for Immuno‐Oncology Research,” Nature Reviews. Clinical Oncology 20, no. 3 (2023): 192–206. 10.1038/s41571-022-00721-2 PMC10593256 36635480

[44] A. G. Gravina , R. Pellegrino , G. Palladino , et al., “ *Hericium erinaceus*, in Combination With Natural Flavonoid/Alkaloid and B(3)/B(8) Vitamins, Can Improve Inflammatory Burden in Inflammatory Bowel Diseases Tissue: An Ex Vivo Study,” Frontiers in Immunology 14 (2023): 1215329. 37465689 10.3389/fimmu.2023.1215329 PMC10350490

[45] A. Tursi , A. D'Avino , G. Brandimarte , et al., “Enhancing Oral 5‐ASA Effectiveness in Mild‐To‐Moderate Ulcerative Colitis Through an *H. Erinaceus*‐Based Nutraceutical Add‐On Multi‐Compound: The “HERICIUM‐UC” Two‐Arm Multicentre Retrospective Study,” Pharmaceutics 16, no. 9 (2024): 1133. 39339171 10.3390/pharmaceutics16091133 PMC11434695

[46] M. Peruhova , D. Miteva , M. Kokudeva , S. Banova , and T. Velikova , “Cytokine Signatures in Inflamed Mucosa of IBD Patients: State‐Of‐The‐Art,” Gastroenterology Insights 15, no. 2 (2024): 471–485.

[47] M. Friedrich , M. Pohin , and F. Powrie , “Cytokine Networks in the Pathophysiology of Inflammatory Bowel Disease,” Immunity 50, no. 4 (2019): 992–1006. 30995511 10.1016/j.immuni.2019.03.017

[48] N. Aslam , S. W. Lo , R. Sikafi , et al., “A Review of the Therapeutic Management of Ulcerative Colitis,” Therapeutic Advances in Gastroenterology 15 (2022): 17562848221138160. 36478780 10.1177/17562848221138160 PMC9720837

[49] X. Song , Q. Zhu , L. Su , et al., “New Perspectives on Migraine Treatment: A Review of the Mechanisms and Effects of Complementary and Alternative Therapies,” Frontiers in Neurology 15 (2024): 1372509. 38784897 10.3389/fneur.2024.1372509 PMC11111892

[50] N. R. West , A. N. Hegazy , B. M. J. Owens , et al., “Oncostatin M Drives Intestinal Inflammation and Predicts Response to Tumor Necrosis Factor–Neutralizing Therapy in Patients With Inflammatory Bowel Disease,” Nature Medicine 23, no. 5 (2017): 579–589. 10.1038/nm.4307 PMC5420447 28368383

[51] P. Ke , B.‐Z. Shao , Z.‐Q. Xu , X.‐W. Chen , and C. Liu , “Intestinal Autophagy and Its Pharmacological Control in Inflammatory Bowel Disease,” Frontiers in Immunology 7, no. 695 (2017): 695. 28119697 10.3389/fimmu.2016.00695 PMC5220102

[52] M. M. Estevinho , C. Rocha , L. Correia , et al., “Features of Fecal and Colon Microbiomes Associate With Responses to Biologic Therapies for Inflammatory Bowel Diseases: A Systematic Review,” Clinical Gastroenterology and Hepatology 18, no. 5 (2020): 1054–1069. 31526845 10.1016/j.cgh.2019.08.063

[53] J. Gubatan , D. R. Holman , C. J. Puntasecca , D. Polevoi , S. J. Rubin , and S. Rogalla , “Antimicrobial Peptides and the Gut Microbiome in Inflammatory Bowel Disease,” World Journal of Gastroenterology 27, no. 43 (2021): 7402. 34887639 10.3748/wjg.v27.i43.7402 PMC8613745

[54] D. H. Kim and J. H. Cheon , “Pathogenesis of Inflammatory Bowel Disease and Recent Advances in Biologic Therapies,” Immune Network 17, no. 1 (2017): 25–40. 28261018 10.4110/in.2017.17.1.25 PMC5334120

[55] W. Zhu , M. G. Winter , M. X. Byndloss , et al., “Precision Editing of the Gut Microbiota Ameliorates Colitis,” Nature 553, no. 7687 (2018): 208–211. 29323293 10.1038/nature25172 PMC5804340

[56] C. Martin‐Gallausiaux , L. Marinelli , H. M. Blottière , P. Larraufie , and N. Lapaque , “SCFA: Mechanisms and Functional Importance in the Gut,” Proceedings of the Nutrition Society 80, no. 1 (2021): 37–49. 32238208 10.1017/S0029665120006916

[57] X.‐F. Zhang , X.‐X. Guan , Y.‐J. Tang , et al., “Clinical Effects and Gut Microbiota Changes of Using Probiotics, Prebiotics or Synbiotics in Inflammatory Bowel Disease: A Systematic Review and Meta‐Analysis,” European Journal of Nutrition 60 (2021): 2855–2875. 33555375 10.1007/s00394-021-02503-5

[58] K. Kannengiesser , C. Maaser , J. Heidemann , et al., “Melanocortin‐Derived Tripeptide KPV Has Anti‐Inflammatory Potential in Murine Models of Inflammatory Bowel Disease,” Inflammatory Bowel Diseases 14, no. 3 (2008): 324–331. 18092346 10.1002/ibd.20334

[59] N. Schlegel , K. Boerner , and J. Waschke , “Targeting Desmosomal Adhesion and Signalling for Intestinal Barrier Stabilization in Inflammatory Bowel Diseases—Lessons From Experimental Models and Patients,” Acta Physiologica 231, no. 1 (2021): e13492. 32419327 10.1111/apha.13492

[60] K. F. Castro‐Ochoa , H. Vargas‐Robles , S. Chánez‐Paredes , et al., “Homoectoine Protects Against Colitis by Preventing a Claudin Switch in Epithelial Tight Junctions,” Digestive Diseases and Sciences 64 (2019): 409–420. 30269272 10.1007/s10620-018-5309-8

