# Supplementary material for: Anti‐Inflammatory Peptides as Promising Therapeutics Agent Against Inflammatory Bowel Diseases: A Systematic Review
Source: JGH Open. 2025 Jun 29;9(7):e70212. doi: 10.1002/jgh3.70212 (PMC12206851; doi:10.1002/jgh3.70212)
Supplement: Supplementary file 1 — Data S1. Supporting Information. [file JGH3-9-e70212-s001.docx]

| **Peptide/Source** | **Observed Effects** | **Research Gaps Identified** |
| --- | --- | --- |
| AVX-470 (Bovine-derived) | Reduced TNF levels, gut inflammation | Need for large-scale human trials |
| Ac2-26 (Annexin A1 mimetic) | Restored gut homeostasis | Pharmacokinetic studies needed |
| AMP-18 (Gastrokine-1) | Stabilized intestinal barrier | Long-term safety evaluation |
| MBCP (Buffalo milk-derived) | Tight junction protection | Mechanistic studies required |
| Hericium erinaceus-derived peptides | Reduced inflammation, clinical benefit | Validation in diverse patient populations |
| Melanocortin peptides (α-MSH analogs) | Immunomodulation, barrier support | Early-phase clinical research |

**Supplementary table 1.** Summary of anti-inflammatory peptides and their research gaps.
